# Supplementary material for: People represent their own mental states more distinctly than those of others
Source: Nat Commun. 2019 May 9;10:2117. doi: 10.1038/s41467-019-10083-6 (PMC6509111; doi:10.1038/s41467-019-10083-6)
Supplement: Supplementary file 1 — Supplementary Information [file 41467_2019_10083_MOESM1_ESM.pdf]

Supplementary Information

Supplementary Methods

Sample sizes for Studies 1 and 2 were determined *a priori* using data and effect sizes from a previous study of similar design [1]. In particular, we targeted the smallest significant effect size (r = .12) from that study. Data from participants in this earlier study were bootstrapped to simulate the drawing of new samples from the population. Sample sizes from 10 to 50 were simulated. Sample sizes of 30 and 35 respectively were estimated to offer 95% power detecting the effects in the earlier study, with the difference in the sample size between the studies due to the differing number and identities of mental states selected. The sample size for Study 3 was based on the sample size for Study 2, with additional participants collected to allow for the potential of attrition from the online experiment. For Study 4, we calculated an expected standardized effect size for the difference between close and far targets (d = .20) using data from Study 3. A parametric power analysis indicated the need for 192 participants to achieve 95% power. We increased our collection target to 350 to allow for the possibility that the effect size estimate from Study 3 was positively biased due to the procedure we used to select states for Study 4.

The far target's biography was comprised of filler material combined with key phrases related to their self-reported politics, religion, hobbies, and college major. It was crafted to be as dissimilar as possible to the participant, as well as being naturally less familiar and less likeable than the self. In Study 1, the distant target's gender was automatically set to match the participant. Names for the distant target were randomly selected amongst the three most common male and female names among college-aged individuals in the United States, so long as that did

not match the participant's own name. In Study 2, the far target's gender was matched to the gender of the close target.

In both imaging studies, participants reported their relationship with each target (e.g. how much they liked them and how close, familiar, and similar they felt to that target), how warm, competent, and extraverted each target was. In Study 2 participants rated how much time, per week, they typically spent with the close target (in person, on the phone, texting, and on social media). In Study 2, participants also rated each of the 25 mental states in the imaging study on three dimensions: valence, social impact, and rationality. In both studies, we also solicited open-ended feedback about the experiment and its purpose.

Participants also provided their demographics and completed several individual difference measures. In Study 1, these measures consisted of the Narcissistic Personality Inventory [2], the Reading the Mind in the Eyes task [3], the MOS social support survey [4], the UCLA Loneliness Scale Version 3 [5], a two-item extraversion measure, a measure of social network size, and the Autism-Spectrum Quotient [6]. In Study 2, participants completed the measures loneliness, extraversion, social network size, and Autism, as well as the Social Interaction Anxiety Scale [7], and a moral judgment questionnaire [8]. These individual difference measures were collected for combination with other data sets in cross-study analyses, and thus we do not report analyses of these data in the present investigation.

Mental states for Study 1 (30 states) and Study 2 (25 states) were selected via simulated annealing algorithms. These algorithms optimized a complex objective functions weighting a number of factors, including the reliability of neural dissimilarity in previous research[1], the correlations between neural similarity and psychological dimensions of mental state representation, and representative, orthogonal sampling along these dimensions. The number of

states in each study was constrained by the planned experimental design and the practical constraints of scanner scheduling. The mental states selected for Study 3 were those which showed the greatest change in distinctiveness as a function of target in Study 2. Similarly, to maximize detection power by maximizing effect size, we selected the states for Study 4 based on those which showed the greatest close-far difference in distinctiveness in Study 3.

For Study 1, we generated a set of 12 image-based scenarios for each state. An initial set of 1234 images was manually collected from the internet. This set was reduced to 24 per state by image analysis, which removed images based on the deviance of their luminance and spatial frequency from the mean of the other images paired with the same state. Four research assistants then coded the images on four features: the number of humans present, the number of non-human characters present, whether the characters (human or otherwise) in the image (if any) were themselves experiencing the mental state in question, and whether the characters were interacting socially. Additionally, independent groups of raters on Amazon Mechanical Turk and from the Princeton University Credit Pool (N = 961) rated the following features of each image: the extent to which the image elicited the appropriate mental states in the rater, the extent to which it would likely elicit the state in another person, abstraction, complexity, appropriateness, likelihood, understandability, and stylistic realism. The final set of images was selected to balance these features across mental states while maximizing the extent to which each image elicited the state in question in other people. In Study 2, text-based scenarios were selected via a previously described genetic algorithm [1], which aimed to select sets of scenarios that maximally evoked each mental state but simultaneously balanced both high- and low-level features of the scenarios across states.

The design of Study 2 grouped targets into blocks during the imaging task. This was done to reduce task demands on the participants, so that they would not have to switch between targets on each trial as in Study 1. However, as a result, the design matrix featured collinearity between mental states within the same target person. This collinearity produced artifactual pattern correlations between some pairs of mental states. Monte Carlo simulation using null data revealed that the artificially induced pattern correlations were approximately equal to the partial correlations between the respective GLM regressors, controlling for the rest of the design matrix. Although the artifactual pattern similarity was necessarily orthogonal to the effect of social distance which we were investigating, we nonetheless wished to mitigate this source of noise. Thus, for all Study 2 analyses involving dissimilarity matrices, we first regressed out a matrix of the partial correlations between regressors of interest. All representational similarity analyses in Study 2 were conducted using the residuals from this regression in place of the raw dissimilarity matrices.

We used multidimensional scaling (MDS) to visualize the similarity between mental states. This technique finds a 2-D configuration, or map, of objects (in this case, mental states) that reproduces a set of measured distances (or dissimilarities) or dissimilarities between said objects. The specific algorithm we used was a nonmetric MDS implemented in the smacof package in R. The MDS mapping allowed us to visualize changes in the size of the mental state representational space as a function of target person in Studies 2 and 3, both of which featured all three targets. For the Study 2 analysis, we computed dissimilarity matrices using state-specific activity patterns from within the voxels which were significant in the Study 1 searchlight analysis (Figure 1). These matrices were averaged across participants prior to MDS. For Study 3, we averaged (and reverse-coded) ratings of pairwise similarity across participants to produce a

single dissimilarity matrix (Figure 3B). The averaged dissimilarity matrices from each study were divided into three parts – one for each target person – and 2-dimensional MDS was applied separately to each target dissimilarity matrix. Procrustes analysis was used to rotate the resulting configurations into a similar orientation to facilitate comparison of targets within each study. These configurations were then plotted together – appropriately scaled to indicate the average dissimilarity in each condition – to illustrate the changing similarity of mental state patterns as a function of social distance. The average dissimilarities between states for each target were plotted as circles in the same space.

In previous research [9], we identified three psychological dimensions – rationality, social impact, and valence – which explained a substantial amount of the neural activity underlying mental state representation. Here we tested whether these dimensions changed in importance as a function of whose mental states participants considered. To do so, we correlated neural pattern similarity between state-specific patterns in Studies 1 and 2, with predictions based on how close the corresponding states were on each of these psychological dimensions, (as well as a fourth dimension from that previous study, termed human mind). Neural similarity was examined within the same feature-selected regions specified above in the primary representational similarity analyses. The analysis was carried out separately for each target person within each participant. Resulting correlations between neural similarity and dimensional proximity were then Fisherized and subjected to paired t-tests (within participant, across target) to determine whether any dimension changed in importance as a function of target person.

In Study 1, we found one significant difference between the self and far target: valence was significantly better predictor of the similarity between one's own states than the similarity between the far target's states (Δr = .07, d = .62, p < .002). This result remained significant

controlling for multiple comparisons across the four dimensions tested. However, this effect did not replicate in Study 2. Indeed, in Study 2, none of the 12 tests (4 dimensions X 3 target comparisons) detected a significant difference in dimensional importance between targets, even prior to controlling for multiple comparisons. Together, the results of these dimensional representational similarity analyses suggest that the shape of the mental state space remains generally consistent across targets, despite the smaller overall size of the space for close and far targets, relative to the self. In other words, the different in the overall granularity of mental state representation between self and other cannot be attributed to a specific reduction in dimensionality along any of the psychological dimensions considered.

Supplementary Figures

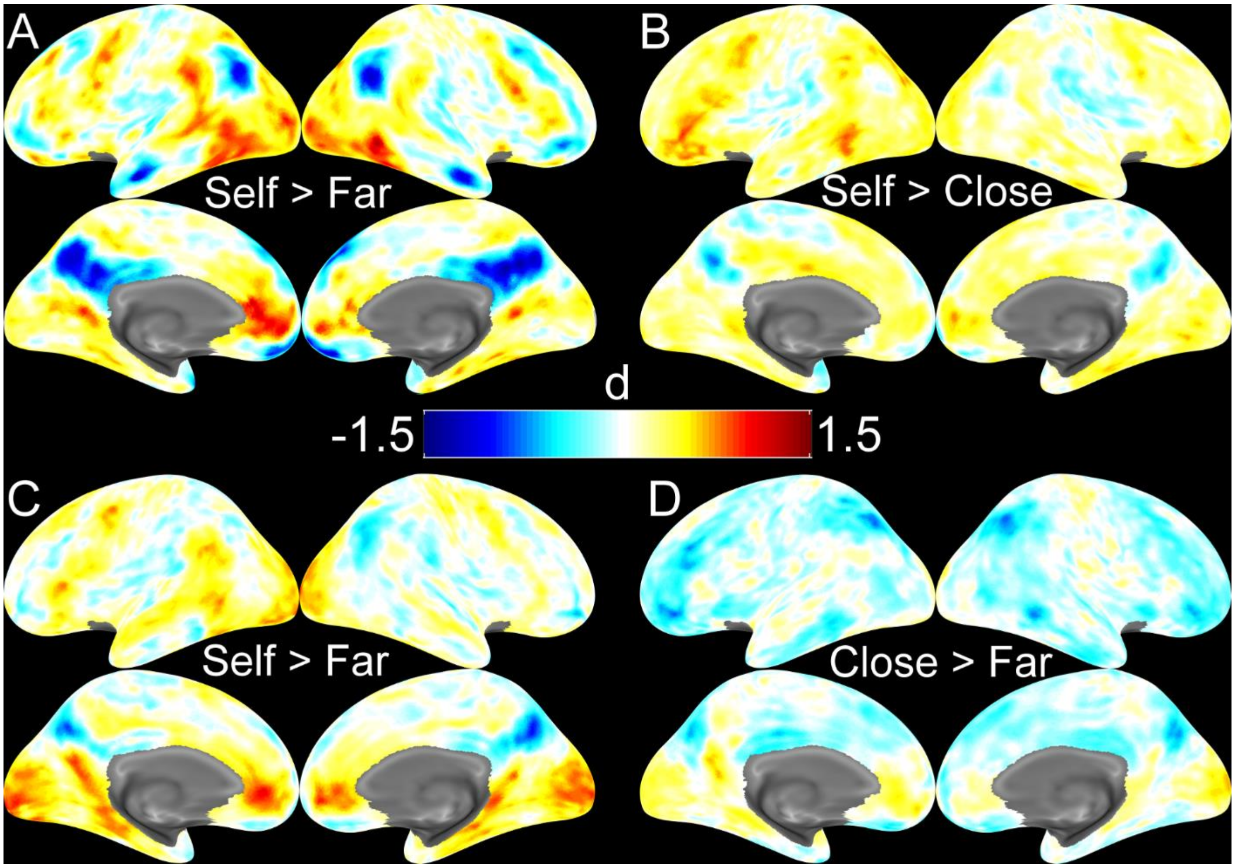

Supplementary Figure 1. *Target effects on univariate activity*. Unthresholded statistical maps reflect the differences between target people in univariate voxelwise activity, collapsing across mental states. Values are presented in terms of standardized effect size (Cohen's *d*). Panel (A) represents the self-other contrast from Study 1; panels (B-D) represent the three pairwise contrast from Study 2.

Supplementary References

1. Tamir, D.I., Thornton, M.A., Contreras, J.M. & Mitchell, J.P. Neural evidence that three dimensions organize mental state representation: Rationality, social impact, and valence. *Proc. Natl. Acad. Sci. USA* **113**, 194-199 (2016).

2. Raskin, R.N. & Hall, C.S. A narcissistic personality inventory. *Psychological reports* (1979).

3. Baron-Cohen, S., Wheelwright, S., Hill, J., Raste, Y. & Plumb, I. The "Reading the Mind in the Eyes" Test revised version: a study with normal adults, and adults with Asperger syndrome or high-functioning autism. *The Journal of Child Psychology and Psychiatry and Allied Disciplines* **42**, 241-251 (2001).

4. Sherbourne, C.D. & Stewart, A.L. The MOS social support survey. *Social science & medicine* **32**, 705-714 (1991).

5. Russell, D.W. UCLA Loneliness Scale (Version 3): Reliability, validity, and factor structure. *J. Person. Assess.* **66**, 20-40 (1996).

6. Baron-Cohen, S., Wheelwright, S., Skinner, R., Martin, J. & Clubley, E. The autism-spectrum quotient (AQ): evidence from Asperger syndrome/high-functioning autism, males and females, scientists and mathematicians. *Journal of autism and developmental disorders* **31**, 5-17 (2001).

7. Mattick, R.P. & Clarke, J.C. Development and validation of measures of social phobia scrutiny fear and social interaction anxiety. *Behav. Res. Ther.* **36**, 455-470 (1998).

8. Young, L., Cushman, F., Hauser, M. & Saxe, R. The neural basis of the interaction between theory of mind and moral judgment. *Proceedings of the National Academy of Sciences* **104**, 8235-8240 (2007).

9. Smith, S.M. & Nichols, T.E. Threshold-free cluster enhancement: addressing problems of smoothing, threshold dependence and localisation in cluster inference. *NeuroImage* **44**, 83-98 (2009).
